# Simultaneous detection of viable *Salmonella* spp., *Escherichia coli*, and *Staphylococcus aureus* in bird's nest, donkey‐hide gelatin, and wolfberry using PMA with multiplex real‐time quantitative PCR

**DOI:** 10.1002/fsn3.2916

**Published:** 2022-05-17

**Authors:** Taobo Liang, Hui Long, Zhongxu Zhan, Yingfei Zhu, Peilin Kuang, Ni Mo, Yuping Wang, Shenghui Cui, Xin Wu

**Affiliations:** ^1^ Jiangxi Institute for Food Control Nanchang China; ^2^ Nanchang Center for Disease Control and Prevention Nanchang China; ^3^ Chengdu Institute of Food and Drug Control Chengdu China; ^4^ National Institutes for Food and Drug Control Beijing China

**Keywords:** homology of medicine and food, mRT‐qPCR, pathogens, viable bacteria detection

## Abstract

*Salmonella* spp., *Escherichia coli*, and *Staphylococcus aureus* are common microbial contaminants within the homology of medicine and food that can cause serious food poisoning. This study describes a highly efficient, sensitive, specific, and simple multiplex real‐time quantitative PCR (mRT‐qPCR) method for the simultaneous detection of viable *Salmonella* spp., *E*. *coli*, and *S*. *aureus*. Primers and probes were designed for the amplification of the target genes *invA*, *uidA*, and *nuc*. Dead bacterial genetic material was excluded by propidium monoazide (PMA) treatment, facilitating the detection of only viable bacteria. This method was capable of detecting *Salmonella* spp., *E. coli*, and *S. aureus* at 10^2^, 10^2^, and 10^1^ CFU/ml, respectively, in pure culture. PMA combined with mRT‐qPCR can reliably distinguish between dead and viable bacteria with recovery rates from 95.7% to 105.6%. This PMA‐mRT‐qPCR technique is a highly sensitive and specific method for the simultaneous detection of three pathogens within the homology of medicine and food.

## Graphical abstract

This study established an efficient, sensitive, specific, and simple multiplex real‐time quantitative PCR (mRT‐qPCR) method for the simultaneous detection of viable *Salmonella* spp., *Escherichia coli,* and *Staphylococcus aureus* in bird's nest, donkey‐hide gelatin, and wolfberry. This method was capable of detecting *Salmonella* spp., *E. coli*, and *S. aureus* at 10^2^, 10^2^, and 10^1^ CFU/ml, respectively, in pure culture. And, the PMA combined with mRT‐qPCR can reliably distinguish between dead and viable bacteria with recovery rates from 95.7% to 105.6%. This study describes a highly efficient, sensitive, specific, and simple multiplex real‐time quantitative PCR (mRT‐qPCR) method for the simultaneous detection of viable *Salmonella* spp., *E. coli*, and *S. aureus*.

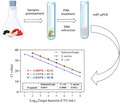

## 1 INTRODUCTION

Bird's nest, donkey hide gelatin, and wolfberry are not only consumed as food but are also used to treat patients in traditional Chinese medicine (Gong et al., 2020; Shan et al., 2015; Wong, 2013). Accordingly, they fall under the theory of the homology of medicine and food. They require extended decoction when used in traditional medicine (Cheung et al., 2021) and can be taken with water or eaten directly as food. However, as foods they are susceptible to microbial contamination during processing, transportation, and storage (Ackerley et al., 2010; Nerín et al., 2016; Otu‐Bassey et al., 2017) and are prone to cause food poisoning. *Salmonella* spp., *Escherichia coli*, and *Staphylococcus aureus* are common pathogenic microorganisms responsible for food poisoning (Elmonir et al., 2021; Kareem & Al‐Ezee, 2020; Soon et al., 2020; Wang et al., 2017). In China, the National Food Safety Standards clearly stipulate acceptable limits for these bacteria and the Chinese Pharmacopoeia defines the relevant regulations. *Salmonella* spp. are the most harmful and must not be detectable. Thus, there is an urgent need to establish a simple, rapid, sensitive, specific, and simultaneous detection method for these three bacteria to diminish the dilemma.

The commonly used method for the detection of pathogenic bacteria is the conventional culture method that identifies targeted bacteria based on whether they can be cultured. However, culturing is complicated and time‐consuming (Kawasaki et al., 2003). Moreover, food samples may contain bacteria that cause competitive inhibition, or dormant or metabolically abnormal bacteria that cannot be successfully cultured, raising the possibility of missed detections and false‐negative results. Molecular biology techniques such as polymerase chain reaction (PCR)‐based methods have been widely used in the detection of pathogenic bacteria. These are simple, rapid, low‐cost, and multifunctional (Xie et al., 2020; Zhang et al., 2018; Zhu et al., 2018). PCR technology includes PCR (Schochetman et al., 1988), fluorescent quantitative PCR (qPCR; Simonetti et al., 1992), and digital PCR (dPCR; Vogelstein & Kinzler, 1999). qPCR monitors the entire reaction process through changes in the intensity of fluorescent signals, enabling real‐time detection and quantification of the cycle threshold (Ct) via a standard curve (Li et al., 2017). The specificity of the TaqMan probe method is higher than the SYBR Green dye method and is capable of multiplex detection via the design of different fluorescent probes (Nejati et al., 2020; Zhang et al., 2015). However, the conventional PCR method cannot distinguish between viable and dead bacteria, which can cause interference leading to false‐positive results (Kim et al., 2015). A novel technique to detect viable bacteria combined PCR with a nucleic acid cross‐linking dye (Nogva et al., 2003). Elimination of the false positives caused by dead cells has been achieved by combining propidium monoazide (PMA) pretreatment with qPCR (Chen et al., 2011). PMA is a nucleic acid cross‐linking agent that selectively enters dead bacteria and binds to genomic DNA, thereby preventing its amplification during PCR. The azido group in any excess PMA reacts with water to produce hydroxylamine, inactivating the PMA (Liang et al., 2019).

In this study, PMA and mRT‐qPCR were combined to simultaneously detect and quantify viable *Salmonella* spp., *E. coli*, and *S. aureus*. Three pairs of primers and probes were designed for the multiplex detection of the target bacteria based on specific genes. Multiplex fluorescent probes were employed in this PMA‐pretreatment qPCR assay for the simultaneous detection of viable bacteria. The applicability of the method was assessed in bird's nest, donkey hide gelatin, and wolfberry samples.

## 2 MATERIALS AND METHODS

### 2.1 Bacterial strains and culture conditions

The bacterial strains used in this study, including seven target bacteria and four non‐target bacteria, are listed in Table 1. The strains were resuscitated in nutrient agar and cultured in brain‐heart infusion at 37℃ (Beijing Land Bridge Technology Ltd.). Suspensions of dead *Salmonella* spp., *E*. *coli*, and *S. aureus* were obtained by heat treating in a metal bath at 80℃ for 10 min, followed by in ice water for 5 min, followed by plate coating to determine that there were only dead bacteria.

**TABLE 1:** Bacterial strains used for specificity detect in this study

No.	Bacterial strains	Source	mRT‐qPCR results		
			*invA*	*uidA*	*nuc*
1	*Salmonella typhimurium*	CMCC 50115	+	−	−
2	*Salmonella paratyphi*	CMCC 80094	+	−	−
3	*Salmonella enteritidis*	ATCC 13076	+	−	−
4	*Escherichia coli*	ATCC 25922	−	+	−
5	*Escherichia coli*	ATCC 8739	−	+	−
6	*Staphylococcus aureus*	CMCC 26001	−	−	+
7	*Staphylococcus aureus*	CMCC 26003	−	−	+
8	*Cronobacter sakazakii*	ATCC 29544	−	−	−
9	*Bacillus cereus*	ATCC 11778	−	−	−
10	*Pseudomonas aeruginosa*	ATCC 9027	−		−
11	*Enterococcus faecalis*	ATCC 29212	−	−	−

Abbreviations: ATCC, American Type Culture Collection; CMCC, Center of Industrial Culture Collection; Results “−”, negative; Results “+”, positive.

### 2.2 Bacterial strains genomic DNA extraction

A simple and rapid boiling method was used to extract bacterial genomic DNA. Pure bacterial culture broth (1 ml) was centrifuged at 12,000 *g* for 3 min, the supernatant discarded, and PBS buffer (0.01 M, pH 7.4) added to resuscitate the cells. This operation was repeated, and the cells suspended in 100 μl ultra‐pure water. This suspension was boiled for 10 min, placed in an ice water bath for 5 min, centrifuged at 12,000 *g* for 3 min, and the supernatant containing the bacterial genomic DNA was stored at −20°C.

### 2.3 Design of primers and probes

Primer Premier 5.0 and Beacon Designer 8 software were used to design specific primers and probes, respectively, based on the genus‐specific gene *invA* (*Salmonella* spp.; Bülte & Jakob, 1995), the species‐specific gene *uidA* (*E. coli*; Kibbee et al., 2013), and the species‐specific gene *nuc* (*S. aureus*; Kim et al., 2001). All primers and probes were determined to be specific by BLAST analysis (National Center for Biotechnology Information). Primers and probes were synthesized by Sangon Biotech (Shanghai, China) to HPLC purification grade. All qPCR processes were performed on a Bio‐Rad CFX96 Touch System. Oligonucleotide sequences of primers and probes are listed in Table 2.

**TABLE 2:** Primers and probe sequence used in this study

Bacterial	Name	Sequences (5′‐3′)
*Salmonella* spp.	*invA*‐F	TTCCGCAACACATAGCCAAGC
	*invA*‐R	AATCCAACAATCCATCAGCAAGG
	*invA*‐p	FAM‐TTTCTCCCCCTCTTCATGCGTTAC‐BHQ1
*E. coli*	*uidA*‐F	CGGAAGCAACGCGTAAACTC
	*uidA*‐R	TGAGCGTCGCAGAACATTACA
	*uidA*‐P	CY5‐CGCGTCCGATCACCTGCGTC‐BHQ‐2
*S. aureus*	*nuc*‐F	CACCTGAAACAAAGCATCCTAAA
	*nuc*‐R	CGCTAAGCCACGTCCATATT
	*nuc*‐P	Texas‐Red‐TGGTCCTGAAGCAAGTGCATTTACGA‐ BHQ1

### 2.4 Optimization of PMA concentration

PMA (1 mg; Beijing BioDee Biotechnology Co. Ltd.) was dissolved in 20% dimethyl sulfoxide (Sinopharm Chemical Reagent Co., Ltd.) at 1 mg/ml and stored at −20°C in darkness. PMA was added to suspensions of dead and viable bacteria (10^8^ CFU/ml) at concentrations of 0, 10, 20, 30, and 40 μg/ml. Suspensions were then incubated in darkness for 5 min, shaken and mixed, then placed on ice around 20 cm from a 500 W halogen lamp for 5 min (Zhou et al., 2017), shaking every 30 s to ensure uniform light exposure. To remove unreacted PMA, cross‐linked samples were centrifuged at 12,000 *g* for 5 min, washed twice with PBS, and re‐suspended in 200 μl ultra‐pure water. Bacterial DNA was extracted for qPCR analysis and optimal PMA concentration was determined based on Ct values. Each qPCR amplification was repeated three times.

### 2.5 mRT‐PCR conditions

mRT‐qPCR was conducted in 25 μl which included 12.5 μl premix Ex Taq (Takara Biotech Co. Ltd.), 2 μl bacterial DNA templates (for each target bacteria), 1.5 μl *invA* primer (10 μM), 0.5 μl *invA* probe (10 μM), 1.0 μl *nuc* primer (10 μM), 0.5 μl *nuc* probe (10 μM), 2.0 μl *uidA* primer (10 μM), and 0.5 μl *uidA* probe (10 μM), 0.5 μl ddH_2_O. The mRT‐qPCR cycling protocol was as follows: 95°C for 5 min (initial denaturation), 40 cycles of 95°C for 5 s (denaturation), and 60°C for 1 min (annealing and extension). The fluorescence signal was acquired during annealing and extension. The annealing temperature was optimized by gradient qPCR and temperature range was set as 55–65°C.

### 2.6 mRT‐qPCR standard curve and limit of detection (LOD)

The target bacteria were cultured overnight, subjected to PMA treatment, and their genomic DNA was extracted and diluted in a 10‐fold gradient (10^8^–10^1^ CFU/ml). Genomic DNA in pure culture medium was analyzed by both simplex and multiplex qPCR. Standard curves were established, correlating Ct values against bacterial concentrations.

**SCHEME 1:**
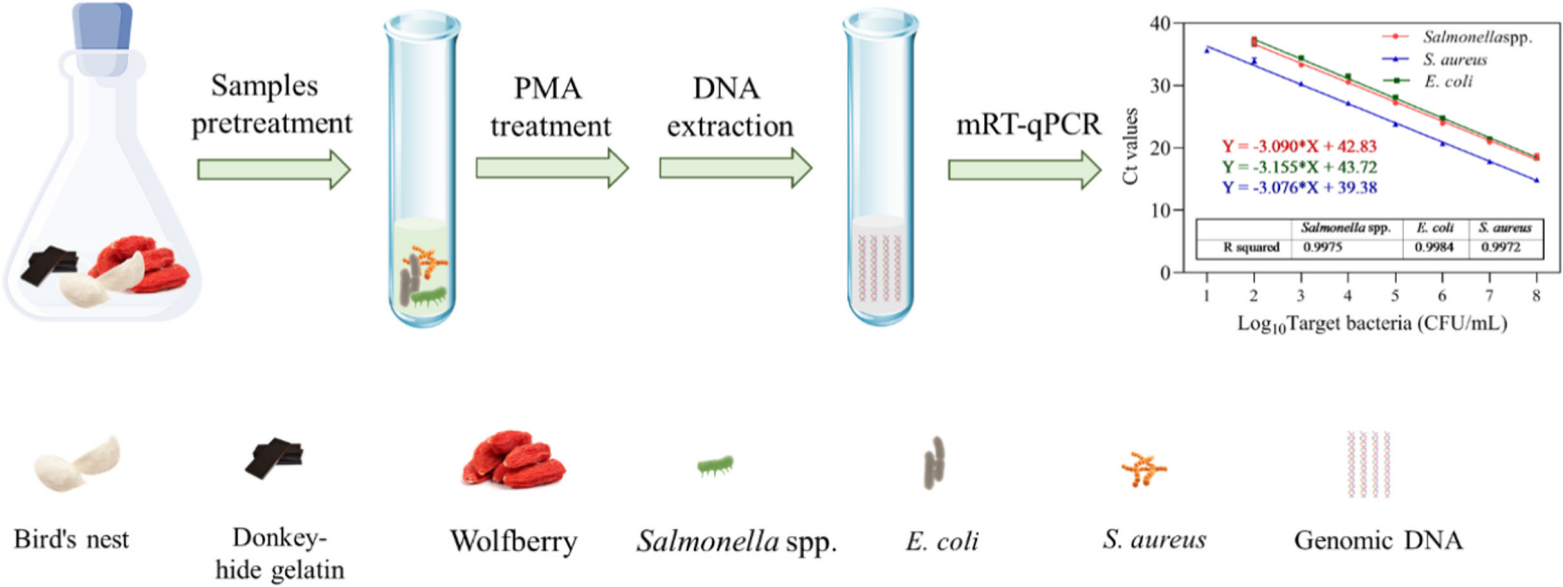
Schematic illustration of PMA‐mRT‐qPCR for simultaneous detection of *Salmonella* spp., *Escherichia coli*, and *Staphylococcus aureus*

To verify the applicability of the PMA‐mRT‐qPCR assay, artificially contaminated common food samples (donkey hide gelatin, bird's nest, and wolfberry) were analyzed. These were purchased from a local drugstore (Drugstore) and 1 g of each food was homogenized with 9 ml PBS. The absence of the three target pathogens was confirmed by culturing. The homogenates were inoculated with *Salmonella* spp., *E*. *coli*, and *S*. *aureus* at 10^1^ to 10^7^ CFU/ml and suspended in 100 μl ultra‐pure water after being treated with PMA. Simplex and multiplex qPCR reactions were optimized for the detection of viable cells in these artificially contaminated samples. All assays were conducted in triplicate in line with Scheme 1 which illustrates the principle and process of PMA‐mRT‐qPCR detection.

### 2.7 Recovery rate

To determine the accuracy of the method, mixed bacterial suspensions containing known numbers of dead and viable bacteria were prepared: 10^8^, 10^6^, 10^4^, and 10^0^ CFU/ml dead bacteria (determined by culture) and 10^7^ CFU/ml viable bacteria. Duplicate groups were treated with PMA and DNA was extracted for mRT‐qPCR determination. Ct values were used for the evaluation of recovery.

### 2.8 Data analysis

All data are expressed as mean ± standard deviation. Data from optimized PMA‐mRT‐qPCR were analyzed by one‐way analysis of variance (ANOVA). Excel (Microsoft Office 2016) was used for statistical analyses. GraphPad Prism 8 and PowerPoint (Microsoft Office 2016) were used to produce figures and diagrams.

## 3 RESULTS AND DISCUSSION

### 3.1 Optimization of PMA‐mRT‐qPCR

**FIGURE 1:**
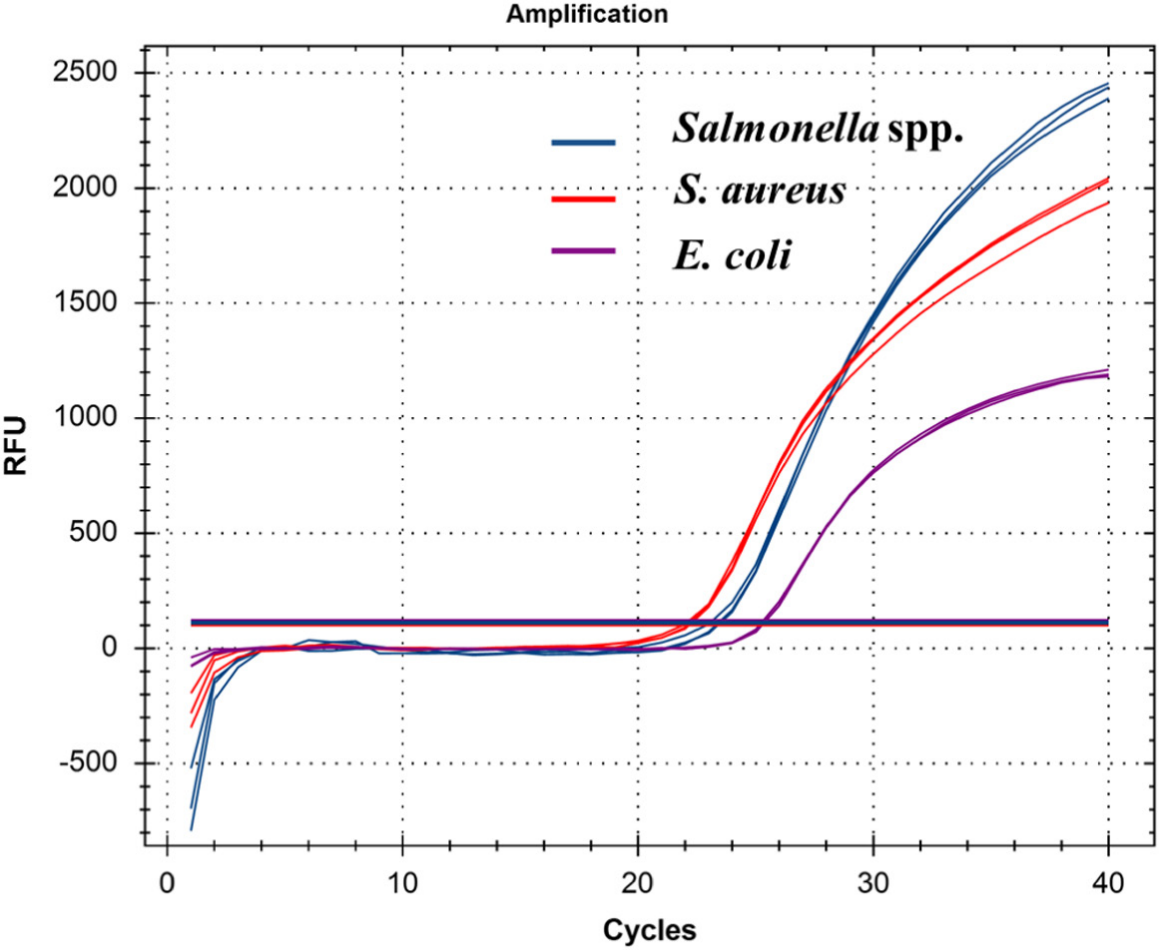
Establishment of mRT‐qPCR system

Primers and probes were designed according to specific genes in the three target pathogenic bacteria and fluorescent groups were modified to achieve simultaneous multiplex detection. As shown in Figure 1, mRT‐qPCR produced three different amplification curves and strong fluorescence signals, indicating that the primers and probes could amplify the target genes simultaneously and accurately.

**FIGURE 2:**
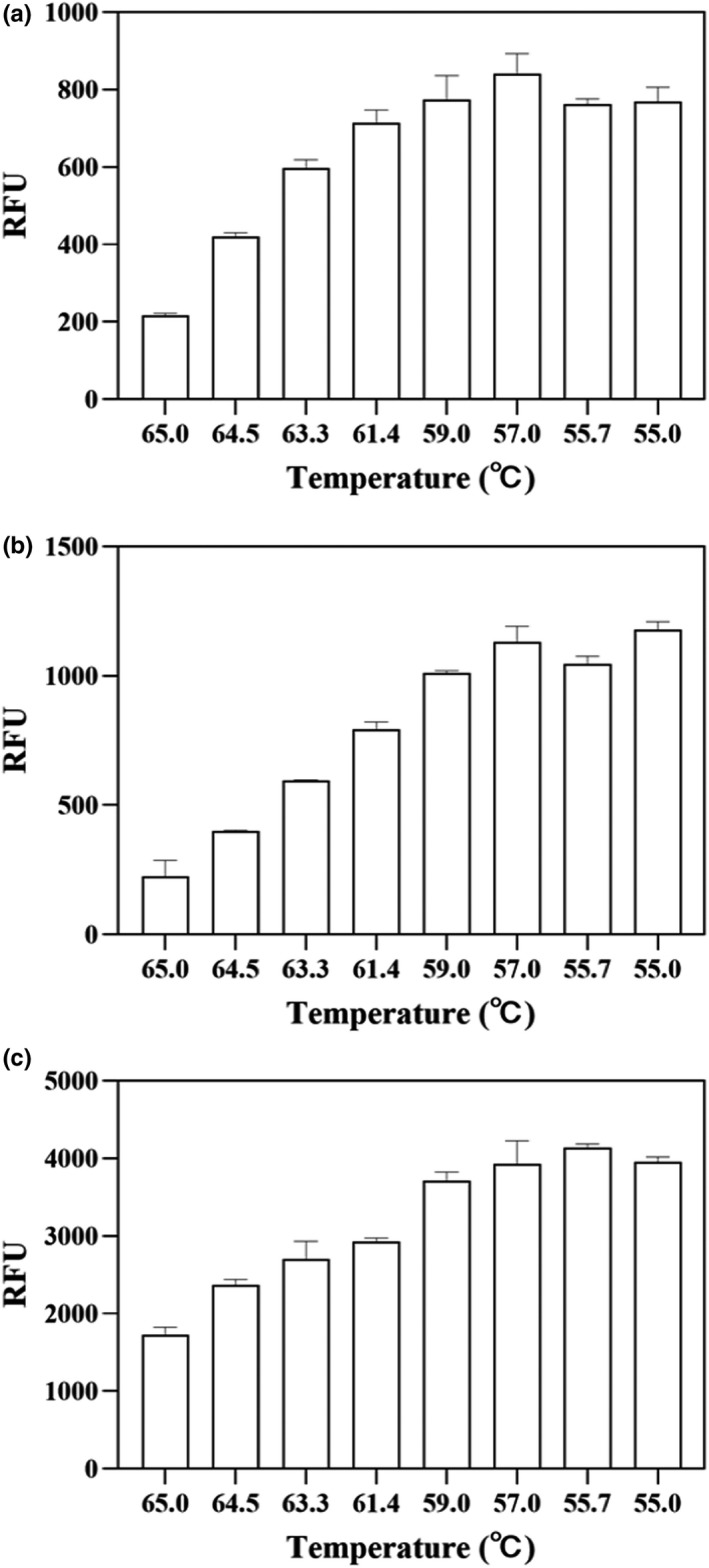
Optimization of annealing temperature. (a) *Salmonella* spp.; (b) *Escherichia coli*; (c) *Staphylococcus aureus*. Each bar represents the average relative fluorescence unit (RFU) of qPCR in triplicates, and error bars indicate standard deviation

The annealing temperature during mRT‐qPCR amplification was optimized. As shown in Figure 2a, the relative fluorescence units (RFU) in the detection of *Salmonella* spp. first increased then decreased as the annealing temperature was increased from 55 to 65°C. RFU peaked at an annealing temperature of 57°C, indicating this was the optimum temperature for the detection of *Salmonella* spp. Similarly, the optimum annealing temperature was 57°C for *E. coli* (Figure 2b) and 55.7°C for *S. aureus* (Figure 2c). An annealing temperature of 57°C was selected to achieve the highest efficiency of multiplex mRT‐qPCR amplification. The three primer pairs have similar optimal annealing temperature values, while the probes have approximately 10°C higher melting temperature values to ensure efficient hybridization to the template (Thornton & Basu, 2011).

**FIGURE 3:**
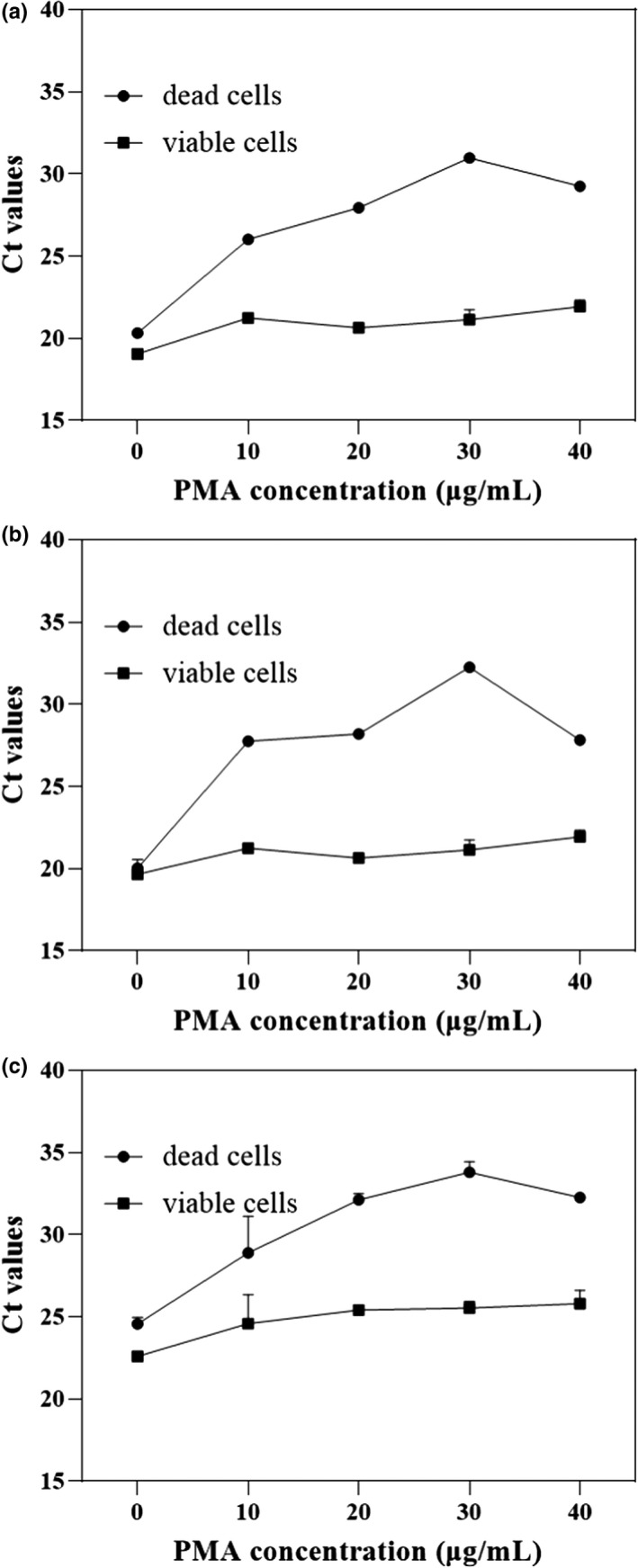
Optimization of PMA treatment concentration. (a) *Salmonella* spp.; (b), *Escherichia coli*; (c) *Staphylococcus aureus*. Each bar represents the average cycle threshold (Ct) values of qPCR in triplicates, and error bars indicate standard deviation

The most appropriate concentration of PMA was determined such that it did not affect DNA amplification of viable bacteria while maximizing the inhibition of DNA detection in dead bacteria. Ct values from dead *Salmonella* spp. increased gradually when the concentration of PMA treatment rose from 0 to 30 μg/ml and decreased when it fell from 30 to 40 μg/ml (Figure 3a). Changes in Ct value of dead *E. coli* (Figure 3b) and *S. aureus* (Figure 3c) treated with PMA followed the same trend, so 30 μg/ml PMA was selected as the optimum.

### 3.2 Simplex and multiplex PMA‐qPCR performance

**FIGURE 4:**
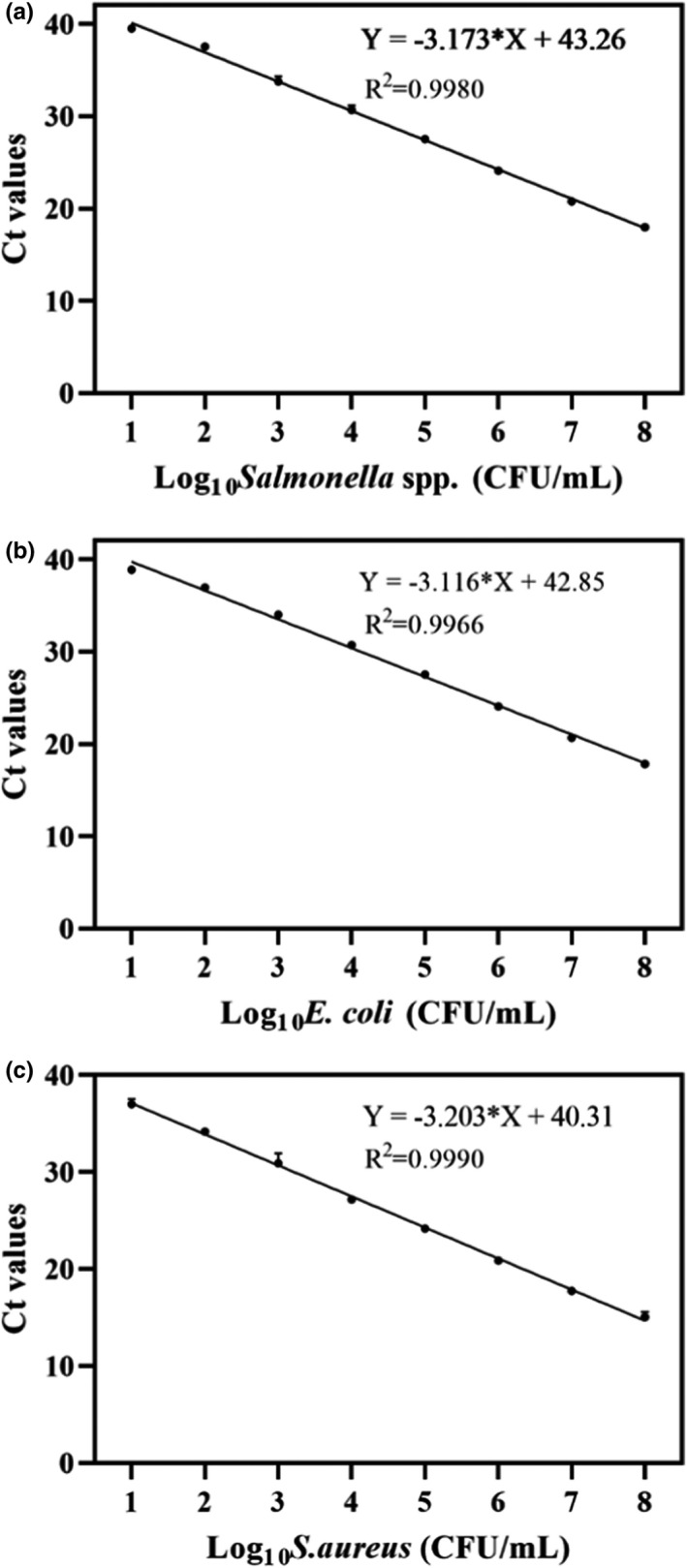
Standard curves and sensitivity of the PMA simplex‐qPCR for different bacterial strains. (a) *Salmonella* spp.; (b) *Escherichia coli*; (c) *Staphylococcus aureus*. Each bar represents the average cycle threshold (Ct) values of qPCR in triplicates, and error bars indicate standard deviation

Simplex PMA‐qPCR was used to evaluate the performance of the method for detecting each species. Figure 4a is the standard curve for a *Salmonella* spp. culture and exhibits a highly linear correlation (*R* ^2^ = .9980) and a maximum amplification efficiency of 106.5% (formula: *E* = (10^−1/slope^−1) × 100%). The standard curve for *E. coli* was highly linear (*R* ^2^ = .9966) with a maximum amplification efficiency of 108.9% (Figure 4b). The standard curve for *S. aureus* was also highly linear (*R* ^2^ = .9990) with a maximum amplification efficiency of 105.1% (Figure 4c). The LOD of the simplex PMA‐qPCR assays for *Salmonella* spp., *E. coli*, and *S. aureus* was 10^1^ CFU/ml. This method showed improvements in amplification efficiency and sensitivity compared with previous studies (Barbau‐Piednoir et al., 2014; Li et al., 2014; Yoon et al., 2018).

**FIGURE 5:**
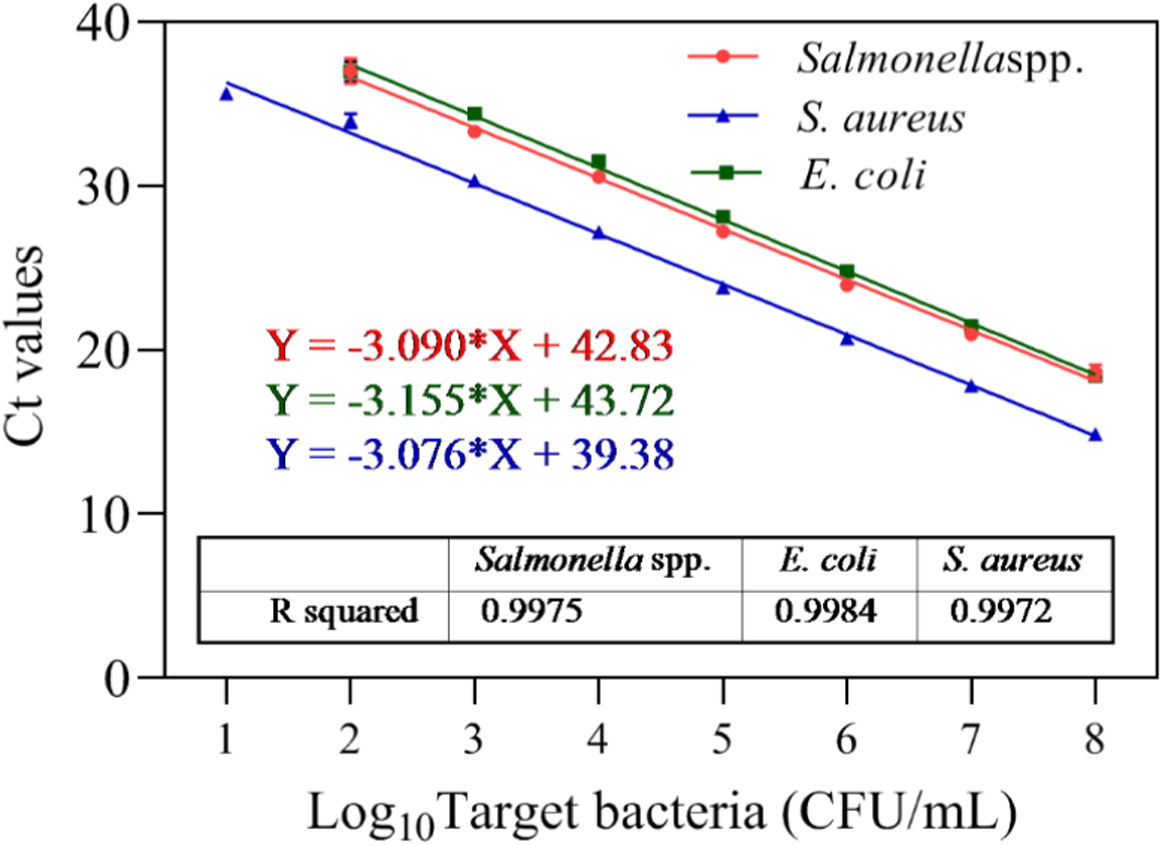
Standard curves and sensitivity of the PMA multiplex‐qPCR for different bacterial strains. Each bar represents the average cycle threshold (Ct) values of qPCR in triplicates, and error bars indicate standard deviation

Standard curves were also established using the optimized PMA‐mRT‐qPCR multiplex assay. Linear correlation was *R* ^2^ = .9975 for *Salmonella* spp., *R* ^2^ = .9984 for *E. coli*, and *R* ^2^ = .9972 for *S. aureus* (Figure 5). The LODs were 10^2^ CFU/ml for *Salmonella* spp. and *E. coli*, and 10^1^ CFU/ml for *S. aureus*. The PMA‐mRT‐qPCR established in this study can simultaneously detect three pathogenic bacteria, and the detection sensitivity was greatly improved compared with previous studies (Forghani et al., 2016), indicating that the primers and probes have great practical application potential.

### 3.3 Evaluation of practical applications

**FIGURE 6:**
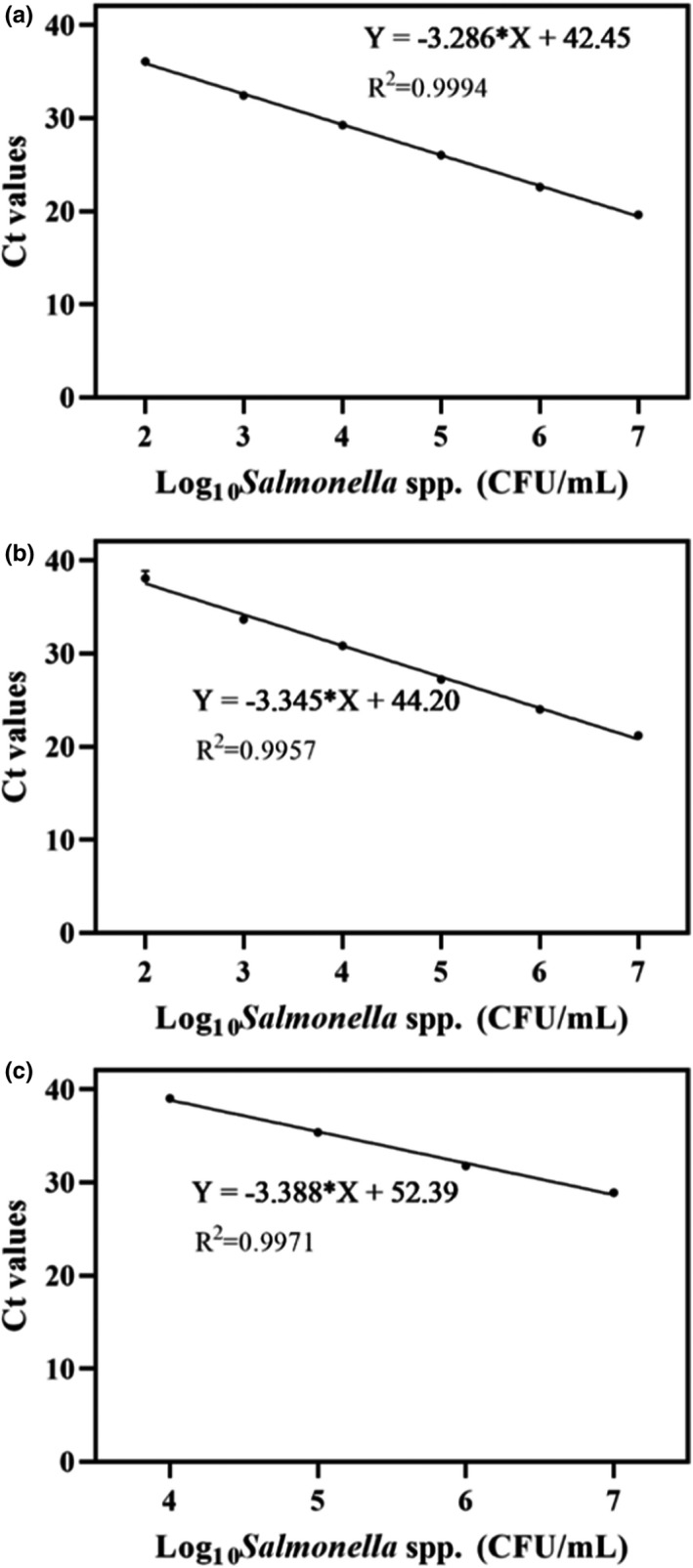
Standard curves and sensitivity of the PMA simplex‐qPCR for *Salmonella* spp. in different artificially contaminated food samples. (a) Donkey‐hide glue; (b) Bird's nest; (c) Wolfberry. Each bar represents the average cycle threshold (Ct) values of qPCR in triplicates, and error bars indicate standard deviation

**FIGURE 7:**
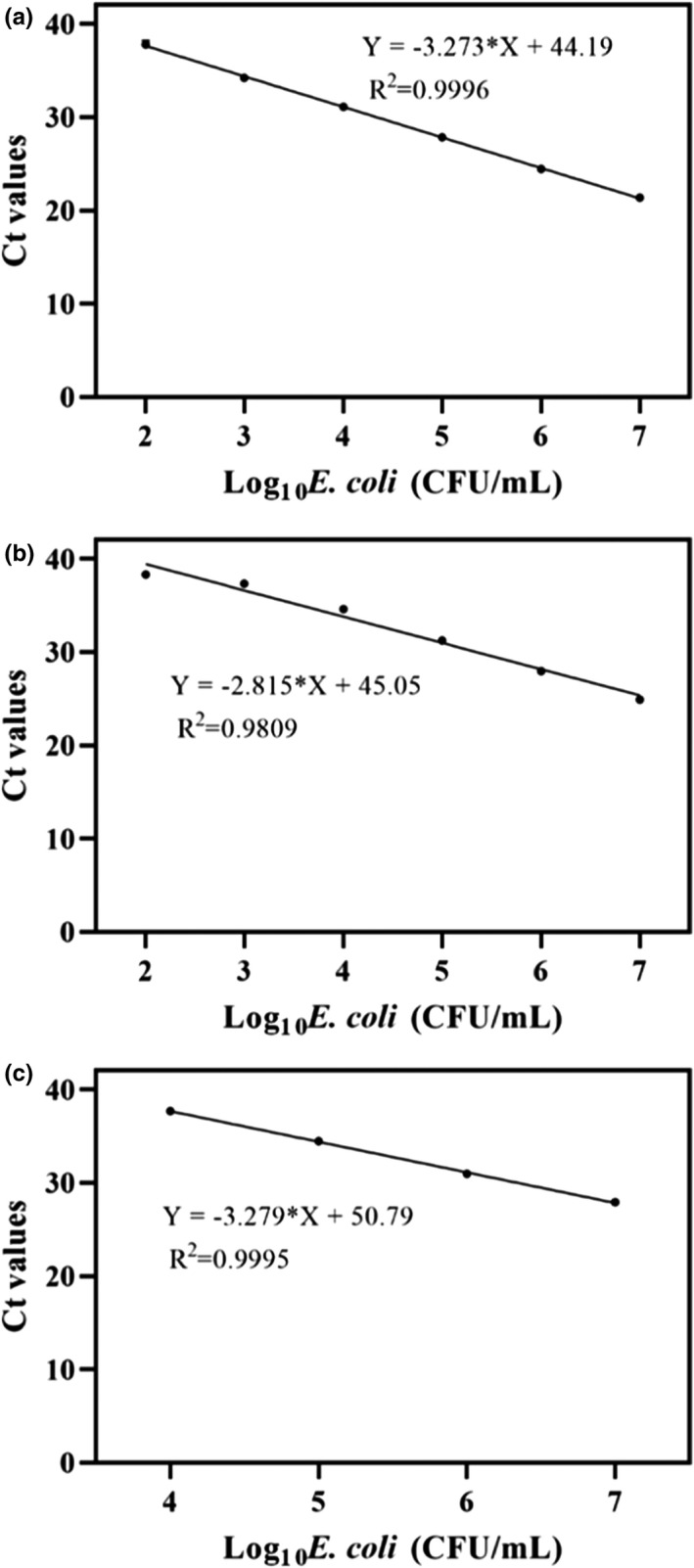
Standard curves and sensitivity of the PMA simplex‐qPCR for *Escherichia coli* in different artificially contaminated food samples. (a) Donkey‐hide glue; (b) Bird's nest; (c) Wolfberry. Each bar represents the average cycle threshold (Ct) values of qPCR in triplicates, and error bars indicate standard deviation

**FIGURE 8:**
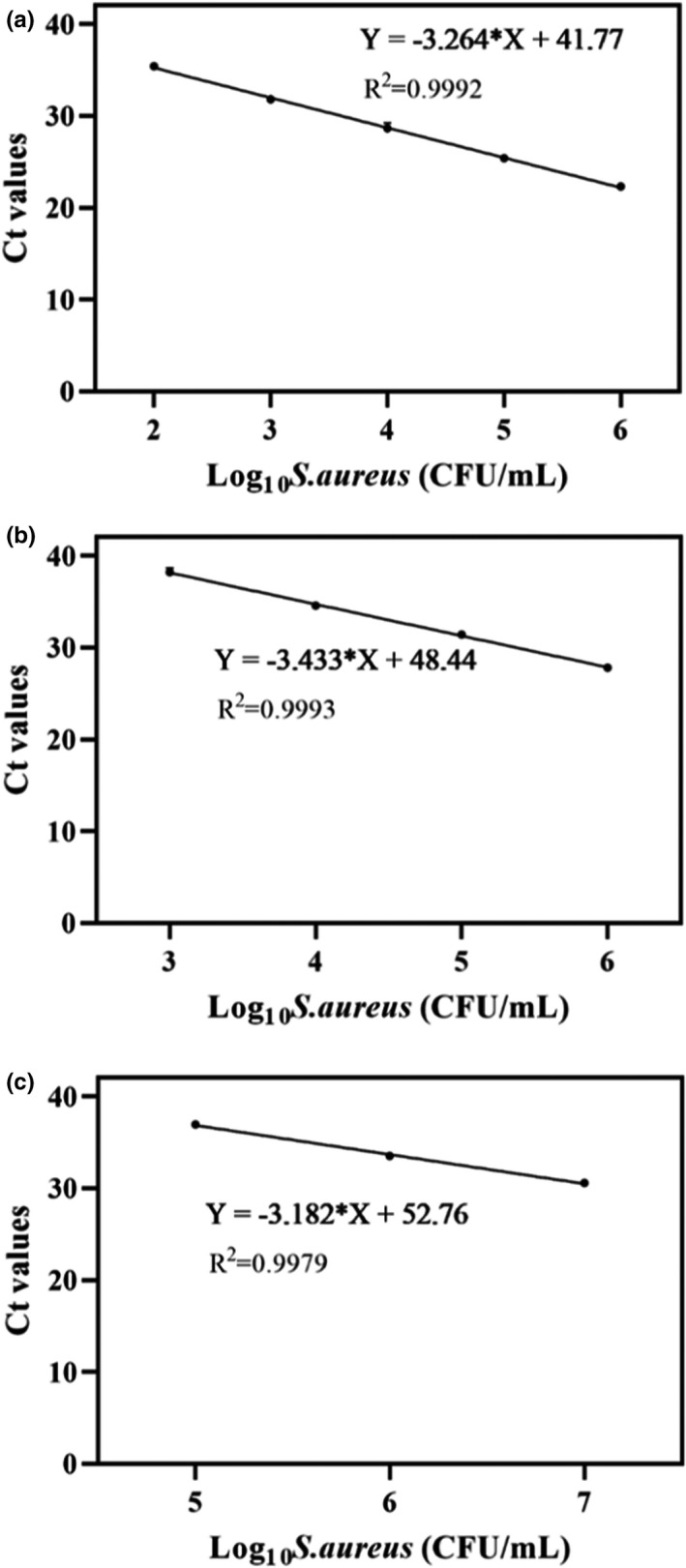
Standard curves and sensitivity of the PMA simplex‐qPCR for *Staphylococcus aureus* in different artificially contaminated food samples. (a) Donkey‐hide glue; (b) Bird's nest; (c) Wolfberry. Each bar represents the average cycle threshold (Ct) values of qPCR in triplicates, and error bars indicate standard deviation

The applicability of this novel PMA‐mRT‐qPCR assay in samples relevant to the homology of medicine and food was demonstrated using artificially inoculated donkey hide gelatin, bird's nest, and wolfberry. Standard curves were established using simplex and multiplex qPCR for each food type. As shown in Figure 6, the linearity and sensitivity of simplex qPCR of *Salmonella* spp. were *R* ^2^ = .9994 and LOD 10^2^ CFU/ml for donkey hide gelatin, *R* ^2^ = .9957 and LOD 10^2^ CFU/ml for bird's nest, and *R* ^2^ = .9971, and LOD 10^4^ CFU/ml for wolfberry. Simplex qPCR of *E*. *coli* produced *R* ^2^ = .9996 and LOD 10^2^ CFU/ml for donkey hide gelatin, *R* ^2^ = .9809 and LOD 10^2^ CFU/ml for bird's nest, and *R* ^2^ = .9995 and LOD 10^4^ CFU/ml for wolfberry (Figure 7). Simplex qPCR of *S. aureus* produced *R* ^2^ = .9992 and LOD 10^2^ CFU/ml for donkey hide gelatin, *R* ^2^ = .9993 and LOD 10^3^ CFU/ml for bird's nest, and *R* ^2^ = .9979 and LOD 10^5^ CFU/ml for wolfberry (Figure 8).

**FIGURE 9:**
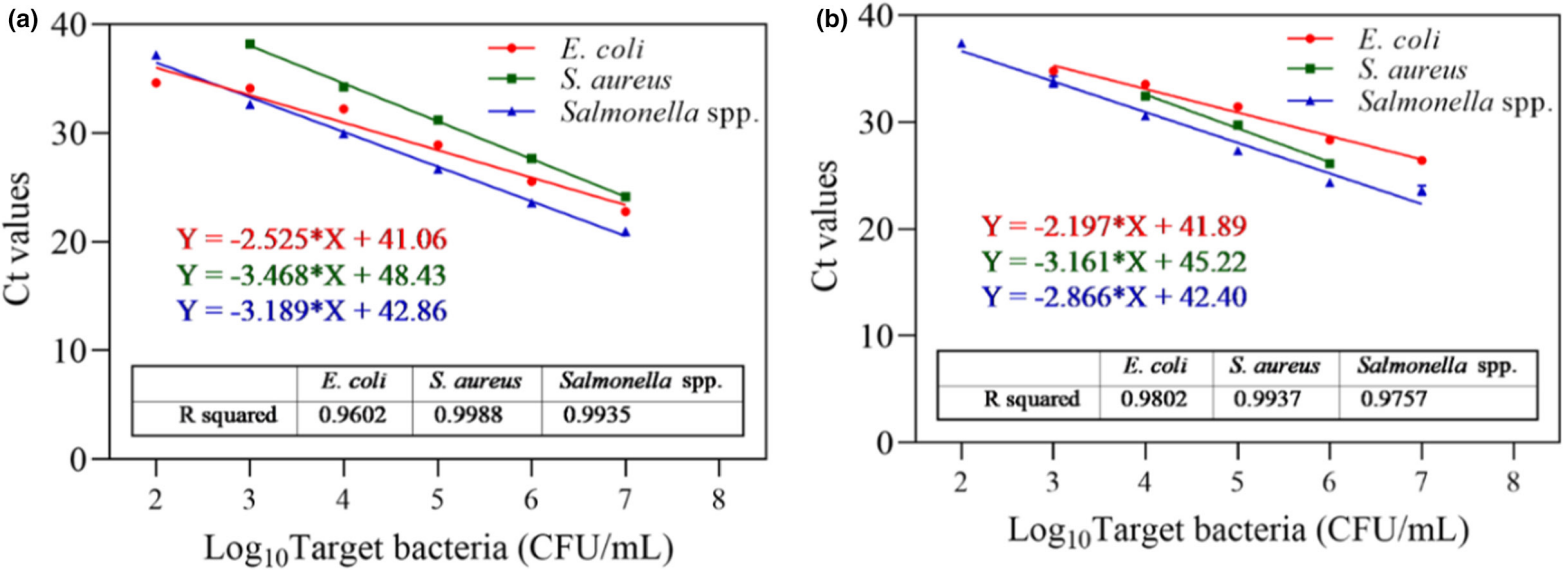
Standard curves and sensitivity of the PMA multiplex‐qPCR for *Salmonella* spp., *Escherichia coli*, and *Staphylococcus aureus* in different artificially contaminated food samples. (a) Donkey‐hide glue; (b) Bird's nest. Each bar represents the average cycle threshold (Ct) values of qPCR in triplicates, and error bars indicate standard deviation

Figure 9 shows the multiplex qPCR standard curves and LODs of the three target bacteria in various food types. All three standard curves exhibited good linear correlations and ranges in donkey hide gelatin (Figure 9a). Detection sensitivity was 10^2^ CFU/ml for *Salmonella* spp. and *E. coli*, and 10^3^ CFU/ml for *S*. *aureus*. Figure 9b shows that *Salmonella* spp. and *E. coli* had good linear ranges in bird's nest, while the *S. aureus* range was narrower. Multiplex LODs were determined to be 10^2^ CFU/ml for *Salmonella* spp., 10^3^ CFU/ml for *E. coli*, and 10^4^ CFU/ml for *S. aureus*. Significantly different LODs and linear ranges were obtained by PMA‐mRT‐qPCR for the three pathogens in different artificially contaminated foods. Notably, standard curves could not be established in wolfberry (data not shown) due to glial interference with DNA extraction. The results show that, compared with other published reports, the practical application of PMA combined with a multiplex detection strategy in the detection of three pathogenic bacteria has higher sensitivity (Elizaquivel & Aznar, 2008).

### 3.4 Recovery of viable bacteria

**FIGURE 10:**
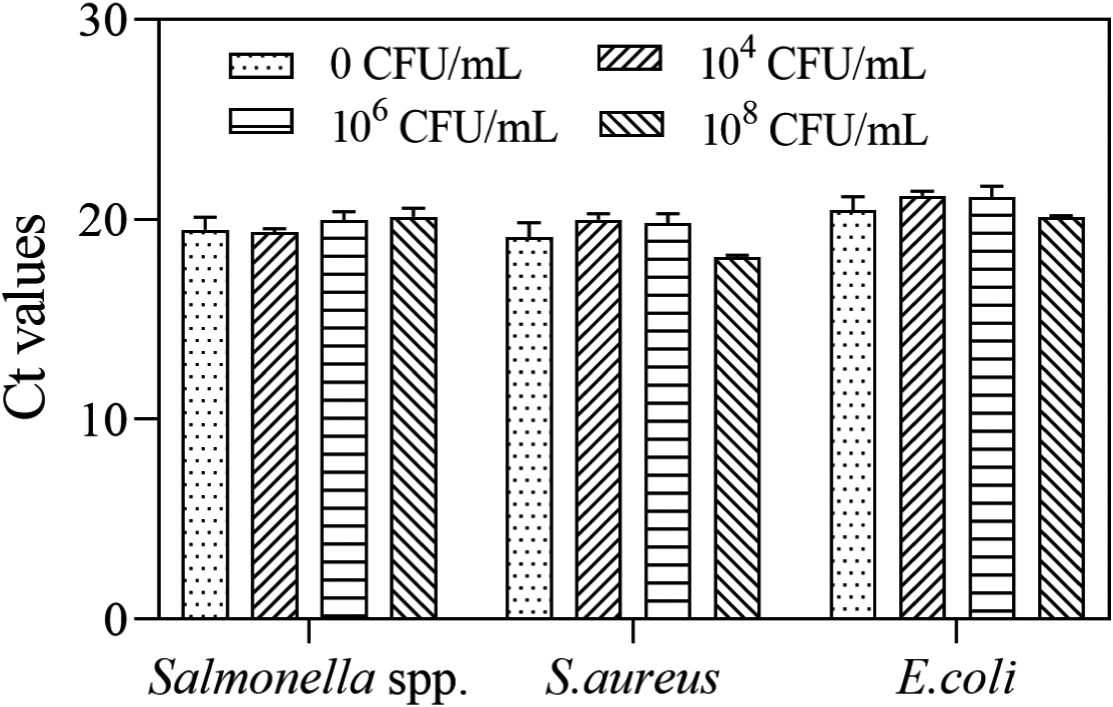
Recovery of viable *Salmonella* spp., *Escherichia coli*, and *Staphylococcus aureus* by PMA‐mRT‐qPCR assay. Each bar represents the average cycle threshold (Ct) values of qPCR in triplicates, and error bars indicate standard deviation

The reliability of the PMA‐mRT‐qPCR assay was determined by testing various concentrations of mixed bacteria (10^8^, 10^6^, and 10^4^ CFU/ml dead bacteria and 10^7^ CFU/ml viable bacteria). Mean Ct values were used to calculate the recovery rate. Recovery of *Salmonella* spp. was 96.7%–100.6%, *E. coli* was 96.6%–101.8%, and *S. aureus* was 95.7%–105.6% (Figure 10). Even in the presence of a high concentration of dead bacteria, this method can accurately determine the concentration of viable bacteria, further illustrating the potential applications of this technique.

### 3.5 Method selectivity

Selectivity of the PMA‐mRT‐qPCR method was assessed by testing 11 bacterial strains, including seven target bacteria (Table 1). Amplification results showed that the *invA* gene can be used to specifically detect strains of *Salmonella* spp., the *uidA* gene can be used to detect strains of *E. coli*, and the *nuc* gene can be used to detect strains of *S*. *aureus*. The results show that the target genes selected in this study have good selectivity, and similar results were found for in previous studies (Baron et al., 2004; Liu et al., 2018; Yanestria et al., 2019).

## 4 CONCLUSION

A rapid, simple, and sensitive method combining PMA and mRT‐qPCR has been developed for the simultaneous detection of viable *Salmonella* spp., *E. coli*, and *S. aureus* in sample types within the homology of medicine and food. Three specific primers and probes were designed for multiplex qPCR amplification to detect the target bacteria. The optimized assay could specifically detect 10^2^ CFU/ml of *Salmonella* spp., 10^2^ CFU/ml of *E*. *coli*, and 10^1^ CFU/ml of *S*. *aureus* in a pure medium. Detection sensitivity differed in various food substrates (bird's nest, donkey hide gelatin, and wolfberry). This method can be used for the safety monitoring of micro‐organisms in medicines and foods, particularly when the abundance of bacteria is limited.

## CONFLICTS OF INTEREST

The authors declare that they have no known competing financial interests or personal relationships that could have appeared to influence the work reported in this paper.

## Data Availability

Research data are not shared.
